# A Short-Term Medical Mission in Rural Nepal: Chief Complaints, Medications Dispensed, and Unmet Health Needs

**DOI:** 10.7759/cureus.15427

**Published:** 2021-06-03

**Authors:** Cindy C Bitter, Carine Dornbush, Cyrus Khoyilar, Charlotte Hull, Heather Elsner-Boldt, Sneedha Mainali, Brian Rice, Errol Visser

**Affiliations:** 1 Emergency Medicine, Saint Louis University School of Medicine, St. Louis, USA; 2 Emergency Medicine, Himalayan Family Healthcare Project, St. Louis, USA; 3 Surgery, Saint Louis University School of Medicine, St. Louis, USA; 4 Anesthesia, Saint Louis University School of Medicine, St. Louis, USA; 5 Family Medicine, Himalayan Family Healthcare Project, St. Louis, USA; 6 Dentistry, The Shine Dental Clinic, Lalitpur, NPL; 7 Emergency Medicine, Stanford University School of Medicine, Palo Alto, USA

**Keywords:** short-term medical mission, nepal, primary care, underserved populations, non-communicable disease

## Abstract

Background

Although Nepal is striving to expand primary health services for its citizens, many remote areas have limited access to basic health care. Short-term medical missions (STMMs) are one way of supplementing human resources for health in underserved areas. This article describes the chief complaints, medications dispensed, and unmet health needs during an STMM in rural Nepal.

Methods

This study is a retrospective analysis of data collected during an STMM that occurred in October 2017. Deidentified data from clinic intake forms were entered into an Excel spreadsheet, and formatted and cleaned. Demographics, chief complaints, medications, and unmet health needs were analyzed using descriptive statistics.

Results

During a two-day health camp, 443 patients were seen. The most common chief complaint was dental (33.4%) followed by musculoskeletal (28.2%) and gastrointestinal (21.2%). Medications were dispensed to 94.8% of patients, primarily analgesics, antibiotics, and ophthalmologic preparations. Of the patients, 21% had unmet health needs, including specialty care and labs or imaging that were beyond the scope of the STMM. One patient was referred urgently to a hospital for treatment of dyspnea and markedly elevated blood pressure.

Conclusion

While STMMs cannot replace access to primary health services, they can provide insight into acute care needs in a system that has limited surveillance. This information describing an acute care patient population should inform future development work.

## Introduction

The Alma-Ata Declaration of 1978 affirmed the human right to health and encouraged member nations to commit to provision of primary health care services, which was identified as key to the attainment of the goal of Health for All [1]. The government of Nepal has made many efforts to expand health services and improve public health indicators since the interim constitution in 2007 recognized the fundamental human right to basic health services. Nepal achieved the United Nations Millennium Development Goals of reducing extreme poverty and child malnutrition, increasing access to universal primary education, improving maternal health, and decreasing morbidity from tuberculosis and malaria [2]. However, significant challenges remain regarding inadequate human resources for health, disparities in access, and budget priorities [3].

Nepal faces multiple challenges in achieving universal access to health care, such as difficult terrain, cultural diversity, and a dichotomy of rich versus poor and urban versus rural. Nepal falls in the low-income group, with a gross national income per capita of $2 per day as well as a total expenditure on health care per capita of $135 (6% of the gross domestic product). This, in effect, means that almost 50% of the population in Nepal is living below the poverty line, where health care expenditure by the individuals would out of necessity be restricted.

Health care in Nepal is provided by a combination of public and private health facilities. Public health facilities include government-supported specialty hospitals, district hospitals, primary health centers, health posts, and community-level resources such as female community health workers and immunization outreach. Private sector health resources include for-profit hospitals and clinics, as well as services provided by international aid organizations and non-government organizations (NGOs). According to the World Health Organization (WHO), government expenditures provide 39.6% of health care funding, while the remaining 60.4% is paid for by individuals when seeking care [4]. The Institute for Health Metrics and Evaluation reports that individual expenditures account for 68.1% of the health budget, with government providing 16.9% and development assistance providing 14.3% [5]. There are some 875 health NGOs operating in Nepal, supplementing the national budget for health by up to 50% [6]. Integration of NGO efforts within the national framework of health care delivery is important to minimize waste and ensure equitable access within the country, but it appears to be suboptimal [7].

Nepal has 0.598 physicians per 1,000 population, compared to the WHO recommendation of 1 per 1000 [8]. However, physicians are concentrated in the Kathmandu valley and other urban areas. One estimate indicates that there are only 0.019 physicians per 1,000 population in rural areas of Nepal [9]. Short-term medical missions (STMMs), locally known as health camps, are one way of providing access in remote regions. This paper describes the patient demographics, chief complaints, medications dispensed, and unmet health needs of a health camp held in rural Nepal. This information may be used to predict training needs for local health workers, as well as pharmaceutical stocks and medical supplies required to meet local acute care needs.

## Materials and methods

Background and study setting

The health camp was located in the village of Thonche in the Manang district. Thonche is located 250 km (155 miles) northwest of the Nepali capital of Kathmandu at an elevation of 1,860 m (6,100 ft). The Manang district is part of the Gandaki Pradesh province, which is located in the center of the country near the Annapurna Conservation Area, along the northern border shared with Tibet. Due to rough terrain and poor road infrastructure, residents have difficulty accessing basic health care. The nearest clinic is in Besisahar, a four- to six-hour trip by an all-terrain vehicle. It takes nine hours to reach Pokhara and 12-15 hours to reach Kathmandu, which have tertiary care services.

The Himalayan Family Healthcare Project is a joint US-Nepali charity dedicated to providing health care in rural Nepal. It conducted a needs assessment and pilot health camp in Manang district in 2010 [10]. Four additional health camps were organized, and a permanent clinic sponsored by the charity opened in July 2019. Ten physicians, seven dentists, two pharmacists, two nurses, and 10 support personnel participated in the health camp.

Study design

This is a retrospective analysis of data from patients who attended a health camp in Manang district in October 2017.

All patients who attended the health camp were eligible to be included in the study, and all provided verbal consent to be included. Data collected on clinic intake forms included demographic information, chief complaint, diagnosis, medications to be dispensed, health counseling, and recommendations for follow-up. Clinic intake forms were completed by physicians and English-speaking Nepali volunteers. Intake forms were deidentified and provided to the study team after care was complete.

Data from the paper clinic forms were entered into an Excel spreadsheet (Microsoft Office 2016, version 16.0, Microsoft Corporation, Redmond, WA) by the research team and was reviewed for accuracy by the lead investigator. Information was verified with members of the data collection team in Nepal as required. Data were analyzed using Microsoft Excel. The health camp was approved by the Ministry of Health of Nepal, which allowed for limited data collection. The Institutional Review Board of Saint Louis University reviewed the protocol and deemed use of deidentified data not to be human subjects research.

## Results

Demographics

A total of 443 patients were assessed in a two-day health camp held in October 2017 in Thonche village (Table 1). Females accounted for 257 (58.0%) visits compared to 112 (25.3%) visits by males. Gender was not specified for 74 (16.7%) patients. Children aged 0-18 years accounted for 86 (19.4%) visits, adults aged 19-65 years accounted for 311 (70.2%) visits, and elders aged 65 years and older accounted for 46 (10.4%) visits. Patients came from a total of 37 villages.

**Table 1:** Demographics

	Number of Patients	Percent of Total
Gender		
Male	112	25.3%
Female	257	58.0%
Not specified	74	16.7%
Age group		
Pediatrics (0-14 years)	73	16.5%
Young adult (15-47 years)	211	47.6%
Middle-aged adults (48-63 years)	109	24.6%
Elderly (64+ years)	50	11.3%

Chief complaints

Dental complaints were the most common problem, as reported by 148 (33.4%) patients (Table 2). Musculoskeletal complaints, including neck pain, sciatica, and osteoarthritis, were reported by 125 (28.2%) patients. Gastrointestinal complaints such as acid-peptic disease, gastroesophageal reflux disease, and diarrhea were reported by 94 (21.2%) patients. Ophthalmologic problems were reported by 50 (11.3%) patients, with ear-nose-throat (ENT) complaints reported by 42 (9.4%). Skin conditions and obstetric-gynecologic complaints were reported by 39 (8.8%) patients each. A small number of patients reported respiratory, cardiac, urinary, and neurologic complaints.

**Table 2:** Chief Complaint Numbers add up to more than 443 as some patients had more than one complaint

Complaints	Number of Patients	Percent of Total
Dental	148	33.4
Musculoskeletal	125	28.2
Gastrointestinal	94	21.2
Ophthalmologic	50	11.3
ENT	42	9.4
Skin	39	8.8
Obstetric-gynecologic	39	8.8

Medications

One or more medication was dispensed to 420 (94.8%) patients. A total of 1443 prescriptions were written, for an average of 3.3 per patient. Also, 74% of medications were from the 2016 National List of Essential Medications for Nepal [11].

Analgesics were dispensed to 368 (83.1%) patients, with non-steroidal anti-inflammatory agents accounting for 247 (67.1%) of all analgesic prescriptions and paracetamol accounting for 121 (32.8%) (Table 3). Antibiotics were prescribed to 217 (48.9%) patients. The most common indication for antibiotics was dental infections/post-extraction prophylaxis, followed by genitourinary complaints, skin infections, and lower respiratory tract infections. Topical ophthalmologic preparations were dispensed to 193 (43.6%) patients, primarily wetting eye drops and topical antibiotics. Gastrointestinal medicines including antacids and proton pump inhibitors were prescribed to 181 (40.8%) patients. Vitamin and mineral supplements were provided to 177 (39.9%) patients. Medications for otolaryngologic complaints such as antihistamines and topical otic antibiotics were provided to 66 (14.8%) patients. Antihypertensive medications were provided to 16 (3.6%) patients, beta-agonist inhalers were provided to 15 (3.4%) patients, and glucose-lowering agents were provided to seven (1.6%) patients.

**Table 3:** Medications by Indication

Medication	Number of Prescriptions	Percent of Total
Analgesics	368	25.2
Antibiotics	217	14.8
Topical ophthalmologic preparations	193	13.2
ENT medications	66	4.5
Gastrointestinal remedies	181	12.3
Vitamins and minerals	177	12.1
Antihelminitics	109	7.5
Antifungals	51	3.5
Antihypertensives	16	1.0
Beta-agonist inhalers	15	1.0
Glucose-lowering agents	7	0.5

Unmet health needs

Unmet health needs have been defined as the difference between necessary care and the care actually received, typically due to barriers related to accessibility, availability, and acceptability [12]. Unmet health needs were present in 92 (21%) patients. One patient was urgently referred to a tertiary center for markedly elevated blood pressure and shortness of breath. Forty-nine (11%) patients required non-urgent specialty services beyond the capability of the health camp, including referral for ophthalmology, complex dental services, obstetrics and gynecology, orthopedics, and neurology. Twelve (2.7%) patients were referred for testing, including chest radiography, ultrasonography, blood work, and electrocardiography. Thirty (6.8%) patients required follow-up that could be managed by a permanent clinic, including repeat blood glucose testing, blood pressure management, wound checks, and reassessment of symptoms.

## Discussion

Data from this health camp provide a snapshot into the acute care health care needs of rural Nepalis and can guide pharmaceuticals and medical supplies required to stock primary health clinics in the region.

The burden of dental disease seen at this health camp was higher than the reported rates of dental complaints in two other studies of health camps in different areas of Nepal [13-14]. Stomach pain (20.1%) and musculoskeletal problems (19.3%) were the most common diagnoses among adults at health camps in Nukawot [13]. Symptomatic complaints such as fever and headache (37.2%) and gastrointestinal complaints (31.9%) were most common in Western Nepal [14]. Neither of these teams included dentists, which may have affected the patients who requested dental services.

STMMs and health camps have been utilized to provide care for patients who would otherwise lack access to primary health services [15]. STMMs are a form of direct assistance by individuals from wealthier nations to persons in low- and middle-income countries, with one survey indicating lifetime participation rates of 32% among US-based physicians and an economic value estimated at US$3.7 billion annually [16]. But STMMs are largely unregulated, and the perceived quality and health outcomes have rarely been subject to scrutiny [17-20]. There are little data to support the effectiveness of one-time health camps in remote areas, but longitudinal projects have potential to reduce the burden of NCDs in the communities they serve [21].

One challenge faced by STMMs is the question of the ranges and types of pharmacological therapies to provide. There may be differences in prescribing patterns between local and foreign volunteer physicians, whether due to lack of awareness of local burden of disease, different antibiotic resistance patterns, or perceived differences in expectations of patients [22]. Inclusion of pharmacists can improve appropriate prescribing on STTM [23]. Given that genetic and environmental influences differ in the communities served, STMM formularies must be carefully considered and planned with local health providers. Use of facility-level data that incorporates burden of disease and capabilities of practitioners can inform pharmacy formularies [24]. In some circumstances, medicines in addition to those listed on the WHO List of Essential Medicines may be appropriate [25]. Understanding the needs of the local population is crucial to providing aid that actually improves health outcomes. Our team dispensed a large number of medications for dental prophylaxis; STMM teams not providing dental services should anticipate dispensing a higher percentage of medications for chronic non-communicable diseases.

Limitations

Health camp providers were a mix of Nepali and foreign providers with extensive experience providing health care in resource-limited settings. However, knowledge of the staffing constraints, lack of follow-up, and formulary limitations may have affected what medications were prescribed and what additional work-up was recommended. The population who presented for treatment at the health camp was self-selected, which likely leads to overestimation of population disease prevalence.

## Conclusions

Patients treated at an STMM in rural Nepal had a variety of infectious and non-communicable disease concerns, with dental problems being the single most common complaint. Analgesics and antibiotics were the most commonly prescribed medications. The majority of complaints could be handled during the STMM. Understanding the acute care needs of the population is important for future deployment of limited health resources.

## References

[1] (2021). Declaration of Alma-Ata. Kazakhstan, USSR.

[2] National Planning Commission (2016). Nepal and the Millennium Development Goals: Final Status Report 2000-2015. Government of Nepal, National Planning Commission, Kathmandu, Nepal.

[3] Regmi K, Naidoo J, Pilkington PA, Greer A (2010). Decentralization and district health services in Nepal: understanding the views of service users and service providers. J Public Health (Oxf).

[4] (2018). Health financing profile 2017 Nepal. http://apps.who.int/iris/bitstream/handle/10665/259643/HFP-NEP.pdf.

[5] (2018). Country Profile: Nepal. http://www.healthdata.org/nepal.

[6] Karkee R, Comfort J (2016). NGOs, foreign aid, and development in Nepal. Front Public Health.

[7] Giri A, Khatiwada P, Shrestha B, Chettri RK (2013). Perceptions of government knowledge and control over contributions of aid organizations and INGOs to health in Nepal: a qualitative study. Global Health.

[8] (2018). Global Health Workforce statistics database. http://www.who.int/gho/health_workforce/physicians_density/en/.

[9] Sengupta A, Zaidi S, Sundararaman T, Onta S, Weerasinghe MC (2018). Tackling the primary care access challenge in South Asia. BMJ.

[10] Lasopa SO, Gunrung States D, Cottler LB (2014). Assessing health needs in rural Nepal. J Health Res.

[11] (2021). Essential Medicines and Health Products Information Portal: A World Health Organization resource. http://apps.who.int/medicinedocs/documents/s23537en/s23537en.pdf.

[12] Pappa E, Kontodimopoulos N, Papadopoulos A, Tountas Y, Niakas D (2013). Investigating unmet health needs in primary health care services in a representative sample of the Greek population. Int J Environ Res Public Health.

[13] Pambos M, Ng J, Loukes J (2012). Demographics and diagnoses at rural health camps in Nepal: cross-sectional study. Fam Pract.

[14] Thapa RK, Thapa P, Parajuli-Baral K, Khan GM (2015). Disease proportions and drug prescribing pattern observed in a free health camp organized at Dhorphirdi Village Development Committee of Western Nepal. BMC Res Notes.

[15] Merali HS, Morgan JF, Uk S (2014). The Lake Clinic - providing primary care to isolated floating villages on the Tonle Sap Lake, Cambodia. Rural Remote Health.

[16] Caldron PH, Impens A, Pavlova M, Groot W (2016). Economic assessment of US physician participation in short-term medical missions. Global Health.

[17] Maki J, Qualls M, White B, Kleefield S, Crone R (2008). Health impact assessment and short-term medical missions: a methods study to evaluate quality of care. BMC Health Serv Res.

[18] Berry NS (2014). Did we do good? NGOs, conflicts of interest and the evaluation of short-term medical missions in Sololá, Guatemala. Soc Sci Med.

[19] Weng YH, Chiou HY, Tu CC, Liao ST, Bhembe PT, Yang CY, Chiu YW (2015). Survey of patient perceptions towards short-term mobile medical aid for those living in a medically underserved area of Swaziland. BMC Health Serv Res.

[20] Nouvet E, Chan E, Schwartz LJ (2018). Looking good but doing harm? Perceptions of short-term medical missions in Nicaragua. Glob Public Health.

[21] Hochheimer C, Khalid M, Vy M, Chang G, Tu D, Ryan M (2017). Addressing noncommunicable disease on short-term medical trips: a longitudinal study of hypertension treatment in Santo Domingo. Ann Glob Health.

[22] Prescott GM, Patzke CL, Brody PM Jr, Prescott WA Jr (2018). Comparison of prescribing patterns between United States and Dominican Republic prescribers on short-term medical mission trips. Int Health.

[23] Clements JN, Rager ML, Vescovi EM (2011). The value of pharmacy services on a short-term medical mission trip: description of services and assessment of team satisfaction. Ann Pharmacother.

[24] Bitter CC, Rice B, Periyanayagam U (2018). What resources are used in emergency departments in rural sub-Saharan Africa? A retrospective analysis of patient care in a district-level hospital in Uganda. BMJ Open.

[25] Broccoli MC, Pigoga JL, Nyirenda M, Wallis L, Calvello Hynes EJ (2018). Essential medicines for emergency care in Africa. Emerg Med J.

